# Pulse-Modulation Eddy Current Evaluation of Interlaminar Corrosion in Stratified Conductors: Semi-Analytical Modeling and Experiments

**DOI:** 10.3390/s22093458

**Published:** 2022-05-01

**Authors:** Zhengshuai Liu, Yong Li, Shuting Ren, Yanzhao Ren, Ilham Mukriz Zainal Abidin, Zhenmao Chen

**Affiliations:** 1 State Key Laboratory for Strength and Vibration of Mechanical Structures, Shaanxi Engineering Research Centre of NDT and Structural Integrity Evaluation, School of Aerospace Engineering, Xi’an Jiaotong University, Xi’an 710049, China; liuzhengshuai@stu.xjtu.edu.cn (Z.L.); renshuting1@stu.xjtu.edu.cn (S.R.); renyanzhao@stu.xjtu.edu.cn (Y.R.); chenzm@mail.xjtu.edu.cn (Z.C.); 2 Leading Edge NDT Technology (LENDT) Group, Malaysian Nuclear Agency, Bangi 43000, Malaysia; mukriz@nuclearmalaysia.gov.my

**Keywords:** electromagnetic non-destructive evaluation, pulse-modulation eddy current technique, semi-analytical model, interlaminar corrosion, defect imaging and evaluation

## Abstract

Interlaminar corrosion (ILC) poses a severe threat to stratified conductors which are broadly employed in engineering fields including aerospace, energy, etc. Therefore, for the pressing concern regarding the safety and integrity of stratified conductors, it is imperative to non-intrusively and quantitatively interrogate ILC via non-destructive evaluation techniques. In this paper, pulse-modulation eddy current (PMEC) for imaging and assessment of ILC is intensively investigated through theoretical simulations and experiments. A semi-analytical model of PMEC evaluation of ILC occurring at the interlayer of two conductor layers is established based on the extended truncated region eigenfunction expansion (ETREE) along with the efficient algorithm for the numerical computation of eigenvalues for reflection coefficients of the stratified conductor under inspection. Based on theoretical investigation, PMEC evaluation of ILC in testing samples are further scrutinized by using the PMEC imaging system built up for the experimental study. The theoretical and experimental results have revealed the feasibility of PMEC for imaging and evaluation of ILC in stratified conductors.

## 1. Introduction

The stratified conductor which consists of a train of thin metallic layers is widely utilized in engineering fields including aerospace, energy, chemical, etc. However, the penetration of moisture and corrosive substances and abrasion of interlaminar surfaces may result in the interlaminar corrosion (ILC) at the interface between each two layers [1]. The fact that ILC is essentially buried within the structure body severely leaves the layered conductor vulnerable to structural failure, since the non-destructive testing methods such as visual testing [2], ultrasonic testing [3], etc., are inapplicable for the inspection and assessment of ILC. Therefore, advanced non-destructive evaluation (NDE) techniques are required to detect and evaluate ILC in order to guarantee the integrity and safety of layered structures in service for an extended period.

In view of the conductive characteristics of stratified conductors, eddy current (EC) testing as well as pulsed eddy current (PEC) testing, which barely requires any contact between the test piece and probe, is one of the preferred NDE techniques for the efficient inspection of conductive structures [4]. In recent years, EC and PEC have been successfully applied to detect the anomalies of railways [5] and delamination in carbon-fiber-reinforced plastic [6], and to estimate the yield strength of ferromagnetic materials [7]. To further improve the testing performance of EC and PEC, the related research is focused on the probe design, signal processing and feature extraction, etc. Wang et al. proposed a non-destructive testing technique integrating EC with PEC to localize the micro-crack in metals and quantitatively characterize its depth separately [8]. Yu et al. designed transverse probes to render the eddy currents perpendicular to circumferential cracks and improved the detection sensitivity to the defects [9]. Bernieri et al. designed a double-coil-based differential probe to detect buried thin cracks with a giant magneto resistance (GMR) sensor [10]. Ge et al. proposed a bobbin probe with two excitation coils to induce more uniform eddy current and pick up signals with array sensors [11]. Besides the novel design of probes, signal features immune to lift-off variations have been investigated. Wang et al. used the dynamic apparent time constant of the PEC-induced coil voltage decay signal to measure wall thinning under the large lift-off variations [12]. Song et al. applied the last peak point of differential PEC signals to measure thickness for non-ferrimagnetic metal under large lift-offs [13]. Zhang et al. proposed Euclidean distances as a signal feature by decoupling interferences of insulations, claddings and the lift-off [14].

As one of the extended NDE methods from EC and PEC, pulse-modulation eddy current (PMEC) testing has been found to be superior to EC and PEC in terms of higher sensitivity and accuracy in detection, imaging and assessment of subsurface defects in planar and tubular conductors [15,16]. The technical advantage of PMEC lies in the fact that the majority of the excitation energy can be effectively allocated to the eddy currents induced right within a conductor body, and thus the dedicated interrogation of the conductor is realized [15]. In a bid to further improve the testing sensitivity, a funnel-shaped PMEC probe has been proposed, and found to be advantageous over the traditional pancake probe in an evaluation of back-surface flaws [17]. Based on this, Yan et al. investigated the methods for imaging of subsurface corrosion in the planar conductor [18], and subsequently established an inversion scheme for the 3D profile reconstruction of detected corrosions [19]. However, to the authors’ knowledge, little research on PMEC inspection of ILC in layered conductors has been conducted.

It is also noticeable that in the aforementioned research, despite the analytical models established based on the extended truncated region eigenfunction expansion (ETREE) modeling [20] for theoretical investigation of PMEC, the corrosion involved in the related model is deemed to be the wall-thinning defect under the assumption that the defect size is appreciably larger than the probe dimension. The scenario regarding the localized corrosion is barely taken into account, leaving a research blank in regard to the theory of PMEC inspection of localized defects and particularly ILC in layered structures. This issue could be tackled in reference to the relevant analytical modeling of EC. Theodoulidis et al. constructed the analytical modeling for calculating eddy currents in a plate with a long slot flaw by the average of an odd and even parity solution [21]. Jiang et al. established an analytical model for eddy current testing of an angled slot with different upper and bottom length in 2D system [22]. Yu et al. proposed an analytical expression for the magnetic field for a cylindrical defect in metal in eddy current testing system by solving the partial differential equations [23]. Tytko et al. presented an axially symmetric mathematical model of an I-cored coil placed over a two-layered conductive material with a cylindrical surface hole [24]. However, more and more complex geometries in analytical models with defect flaws make the numerical implementation more difficult [25]. To solve this problem, some researchers tried to develop more efficient calculation approach to solve the analytical modeling. The main obstacle for implementation of the analytical model is solving discrete complex eigenvalues of the partial differential equations in a defect domain. To find eigenvalues in a defect domain, Theodoulidis et al. used the Newton–Raphson algorithm [21] and a derived Cauchy principle [26] developed by Delves and Lyness [27] for the computation of eigenvalues. Darko et al. further improved Delves and Lyness’ work and designed an iteration algorithm for dividing regions in complex planes for guaranteeing the efficient solution of eigenvalues [28]. Strakova adapted Cauchy’s residue theorem and presented a contour integral method for the localization of eigenvalues of a matrix pencil in a bounded domain in the complex plane [29]. Tytko proposed a method for the multilevel computation of complex eigenvalues by combining both the Newton method and Cauchy principle and applying them in different regions of the complex plane [25]. The related analytical modeling of EC with localized defects could be supportive of the establishment of theoretical models for PMEC evaluation of ILC in stratified conductors.

In a bid to scrutinize the feasibility of PMEC for the evaluation of ILC in stratified conductors, a series of theoretical simulations of PMEC testing of localized ILC with different radii and depths are carried out with a semi-analytical model established based on the ETREE modeling. In parallel, a PMEC system has been built up for experimental investigation in a bid to further confirm the applicability of PMEC for the detection, imaging and assessment of ILC. The rest of the paper is organized as follows: Section 2 elaborates the semi-analytical modeling of PMEC for ILC evaluation. The theoretical and experimental investigations involving characteristics and features of PMEC responses to ILC, etc., are intensively presented in Section 3 and Section 4, respectively. The investigation results are summarized and concluded in Section 5.

## 2. Semi-Analytical Modeling Regarding PMEC Evaluation of ILC

### 2.1. Field Formulation

**Figure 1:**
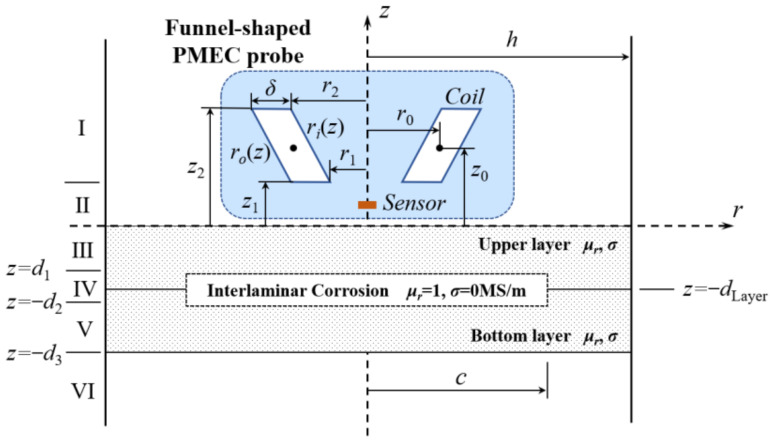
A 2D axisymmetric model of a funnel-shaped PMEC probe placed over a two-layer stratified conductor with an ILC at the interface between the upper and bottom layers.

In an effort to investigate PMEC evaluation of ILC in stratified conductors, a semi-analytical model based on ETREE modeling is established. A 2D axisymmetric model of PMEC evaluation of a two-layer conductor subject to an ILC is portrayed in Figure 1. The model comprises a funnel-shaped PMEC probe and a two-layer conductor. The funnel-shaped probe consists of: (1) an excitation coil with the parallelogram cross-section for generation of the incident magnetic field; and (2) a solid-state magnetic-field sensor which is deployed at the bottom center of the excitation coil and used for acquiring the testing signal of the net magnetic field (superposition of the incident and eddy-current-induced fields). It is noted that the ILC residing at the interface between the upper and bottom layers is in the shape of a cylinder with the radius of *c* and depth/thickness of *D*, *D* = *d*_2_ − *d*_1_. The region of interest (ROI) truncated with the radial distance of *h* in Figure 1 is divided into a series of sub-regions numbered from Region II to Region VI in reference to the continuity of electromagnetic field over the boundaries of the coil, conductor and ILC with the *z* coordinates of *z*_1_, 0, −*d*_1_, −*d*_2_ and −*d*_3_.

Based on ETREE modeling for transient eddy current testing [15], the closed-form expression of *z*-component of the transient net magnetic field which is sensed by the sensor at an arbitrary coordinate of (*r*, *z*) in Region II can be written as:

(1) $$B_{z}\left(r,z,t\right)=I\left(t\right)⊗F^{-1}\left[B_{z}\left(r,z,\omega \right)\right],$$

where *I*(*t*) denotes the excitation signal of the electric current driving the coil. ⊗ stands for the circular convolution. *F*^−1^ denotes the inverse Fourier transform. *B_z_* (*r*, *z*, *ω*) is the spectral response with respect to every harmonic at an angular frequency of *ω* within the excitation signal. For each harmonic, *B_z_* (*r*, *z*, *ω*) can be written in a form of a double integral of the *z*-component of the total magnetic field resulted from a filament excitation coil (with the radius of *r*_0_ and liftoff of *z*_0_), i.e., $B_{z}^{f}$ (*r*, *z*, *ω*) as:

(2) $$B_{z}\left(r,z,\omega \right)=\frac{N_{coil}}{\delta \left(z_{2}-z_{1}\right)}\int_{z_{1}}^{z_{2}}\int_{r_{i}\left(z\right)}^{r_{o}\left(z\right)}B_{z}^{f}\left(r,z,\omega \right)drdz,$$

where *N_coil_* is the number of turns of the excitation coil. *δ* denotes the coil radial thickness. *z*_1_ and *z*_2_ are the *z*-integral limits corresponding to the lower and upper boundaries of the coil. *r_i_* (*z*) and *r_o_* (*z*), which stand for the inner and outer boundaries of the coil, respectively, are taken as the lower and upper limits for the *r*-integral. They are written as:

(3) $$\left\{\begin{array}{l} r_{i}(z)=r_{1}+\frac{\delta z}{z_{2}-z_{1}}\\ r_{o}(z)=r_{1}+\delta +\frac{\delta z}{z_{2}-z_{1}} \end{array}\right..$$

It is noteworthy that since the localized ILC is taken into account in the model, in Equation (2) $B_{z}^{f}$(*r*, *z*, *ω*) can hardly be expressed in the form of series expansion, but is formulated in the matrix notation. Since the magnetic field can be derived from the identity, i.e., the curl of the magnetic vector potential, in the cylindrical coordinate system it is thus written as:

(4) $$\;{\vec{B}}^{f}(r,z,\omega )=-\frac{\partial A^{f}(r,z,\omega )}{\partial z}\vec{r}+\frac{1}{r}\frac{\partial [rA^{f}(r,z,\omega )]}{\partial r}\vec{z},$$

where $\vec{r}$ and $\vec{z}$ are the unit vectors. *A^f^* denotes the magnetic vector potential resulting from the filament excitation coil. Referring to [23], it is expressed as:

(5) $$\left\{\begin{array}{l} A^{f}(r,z,\omega )=J_{1}(\kappa^{T}r)\left(e^{\kappa z}C+e^{-\kappa z}D\right)\\ C=\frac{\mu_{0}r_{0}}{2}e^{-\kappa z_{0}}\kappa^{-1}E^{-1}J_{1}(\kappa r_{0})\\ D=\Gamma C \end{array}\right.,$$

where *J*_1_ (**κ***^T^r*) and *J*_1_ (**κ***r*_0_) represent the 1 × *N_s_* and *N_s_* × 1 matrices with the corresponding elements of *J*_1_ (*κ_i_r*) and *J*_1_ (*κ_i_r*_0_), respectively. *J_n_* is the first-kind Bessel function of the order of *n*. *e***^κ^***^z^* and *e****^−^*^κ^***^z^* denote the *N_s_* × *N_s_* diagonal matrices with the individual elements of $e^{\kappa_{i}z}$ and $e^{-\kappa_{i}z}$, respectively. *μ*_0_ is the permeability of vacuum. **C** and **D** are *N_s_* × 1 matrices. Based on Equations (4) and (5), the magnetic field at the sensor position is formulated as:

(6) $${\vec{B}}^{f}(r,z,\omega )=-J_{1}(\kappa^{T}r)\kappa \left(e^{\kappa z}C-e^{-\kappa z}D\right)\vec{r}+J_{0}(\kappa^{T}r)\kappa \left(e^{\kappa z}C+e^{-\kappa z}D\right)\vec{z}.$$

In Equations (5) and (6), **κ** is a diagonal matrix with the diagonal elements of *κ_i_*, *i* = 1, 2, 3…*N_s_*. *κ_i_* are eigenvalues for the air domains, which are the positive roots of the equation:

(7) $$J_{1}(\kappa_{i}h)=0.$$

**E** is a diagonal matrix with the elements expressed as:

(8) $$E_{ii}=\frac{h^{2}J_{0}^{2}(\kappa_{i}h)}{2}.$$

**Γ** is a *N_s_* × *N_s_* full matrix representing the conductor reflection coefficient. It can be derived from the boundary conditions implying the continuity of electromagnetic field over each interface in the domain under the excitation coil (i.e., Regions II, III,…and VI), and thus written as:

(9) $$\Gamma =\;K_{2}^{-1}K_{1},$$

where **K_1_** and **K_2_** are formulated as:

(10) $$\left\{\begin{array}{l} K_{1}=\left(L_{1}M_{1}-L_{2}M_{2}\right)Ee^{-\lambda d_{1}}N_{1}-\left(L_{2}M_{1}-L_{1}M_{2}\right)Ee^{\lambda d_{1}}N_{2}\\ K_{2}=\left(L_{2}M_{1}-L_{1}M_{2}\right)Ee^{\lambda d_{1}}N_{1}-\left(L_{1}M_{1}-L_{2}M_{2}\right)Ee^{-\lambda d_{1}}N_{2} \end{array}\right..$$

It is noted that **L_1_**, **L_2_**, **M_1_**, **M_2_**, **M_3_**, **M_4_**, **N_1_** and **N_2_** in Equation (10) are the coefficient matrices which are expressed as:

(11) L1=N1−1eλd2−d 3E−1M3−N2−1eλd3−d2E−1M4epd1−d2L2=N2−1eλd3−d2E−1M3−N1−1eλd2−d3E−1M4epd2−d1,

(12) $$\left\{\begin{array}{l} M_{1}=U^{-1}+{\left(Up\right)}^{-1}\lambda ,\;\;\;\;M_{3}=U+\lambda^{-1}Up\;\;\\ M_{2}=U^{-1}-{\left(Up\right)}^{-1}\lambda ,\;\;\;\;M_{4}=U-\lambda^{-1}Up \end{array}\right.,$$

(13) $$\left\{\begin{array}{l} N_{1}=I+\lambda^{-1}\kappa \\ N_{2}=I-\lambda^{-1}\kappa \end{array}\right..$$

In these expressions, **I** denotes the *N_s_* × *N_s_* identity matrix. **λ** is the *N_s_* × *N_s_* diagonal matrix with the diagonal elements of *λ_i_* which are eigenvalues for the layered conductor free of ILC, and can be expressed as:

(14) $$\lambda_{i}=\sqrt{\kappa_{i}^{2}+j\omega \mu_{0}\mu_{r}\sigma },$$

where *σ* and *μ_r_* denote the apparent conductivity and relative permeability of the conductive layers, respectively. The elements *U_ij_* in the *N_s_* × *N_s_* full matrix **U** can be computed out by using the equation written as:

(15) $$U_{ij}=\frac{-ck^{2}}{\left(p_{j}^{2}-\kappa_{i}^{2})(q_{j}^{2}-\kappa_{i}^{2}\right)}\left[\kappa_{i}J_{0}(\kappa_{i}c)J_{1}(p_{j}c)-p_{j}J_{1}(\kappa_{i}c)J_{0}(p_{j}c)\right]R_{1}(q_{j}c),$$

where *k*^2^ = *jωμ*_0_*μ_r_σ*. *R_n_* (*q_i_c*) is formulated as:

(16) $$R_{n}(q_{i}c)=Y_{1}(q_{i}h)J_{n}(q_{i}c)-J_{1}(q_{i}h)Y_{n}(q_{i}c),$$

where *Y_n_* denotes the second-kind Bessel function of the order of *n*. The diagonal elements *p_i_* in the *N_s_* × *N_s_* diagonal matrix **p** are eigenvalues for the ILC region. They can be sought by finding the roots of the equation:

(17) $$p_{i}J_{0}\left(p_{i}c\right)R_{1}\left(q_{i}c\right)-q_{i}J_{1}\left(p_{i}c\right)R_{0}\left(q_{i}c\right)=0.$$

In Equations (15)–(17), *q_i_* is also the eigenvalues for the ILC domain, and can be derived by using the equation:

(18) $$q_{i}=\sqrt{p_{i}^{2}-j\omega \mu_{0}\mu_{r}\sigma }.$$

By using Equations (1) and (2), the temporal responses of PMEC to localized ILC at the interface of the layered conductor can be readily predicted. 

### 2.2. Numerical Calculation of Eigenvalues

The calculation of eigenvalues particularly *p_i_* is a fundamental step in predicting the PMEC signals via the semi-analytical model. In this paper, eigenvalues in the ILC domain are acquired based on the Delves and Lyness algorithm calculating the eigenvalues and further improving the precision by Newton–Raphson iteration. When applying the Delves and Lyness algorithm to seek eigenvalues, the roots of Equation (17) are equivalent to zeros of the function written as:

(19) $$f\left(x\right)=xJ_{0}\left(xc\right)R_{1}\left(qc\right)-qJ_{1}\left(xc\right)R_{0}\left(qc\right).$$

According to the Cauchy argument principle developed by Delves and Lyness, the summation of *n*^th^ power of zeros of an analytic function enclosed by the contour *C* in the complex plane can be expressed as:

(20) $$s_{n}=\frac{1}{2\pi j}\oint_{C}x^{n}\frac{f^{\prime }(x)}{f(x)}dx=\overset{N}{\underset{i=1}{\sum }}\xi_{i}^{n}\;\;\;\;n=1,2,\ldots N,$$

(21) $$s_{0}=\frac{1}{2\pi j}\oint_{C}x^{0}\frac{f^{\prime }(x)}{f(x)}dx=\overset{N}{\underset{i=1}{\sum }}\xi_{i}^{0}=N,$$

where *f’*(*x*) is the derivative function of *f*(*x*). *ξ_i_* are zeros of the *f*(*x*) enclosed by the contour *C*, giving the solutions to *p_i_* in Equation (17). By dividing the complex plane into a series of discretized subareas, information of eigenvalues in each subarea is derived from Equations (20) and (21). In each subarea, the number of zeros *N* is equal to *s*_0_. To obtain to solutions to *p_i_* in defect domain, it is required to solve the *N*^th^ degree system of equations which is written as:

(22) $$\left\{\begin{array}{l} s_{1}=p_{1}+p_{2}+\ldots +p_{N}\\ s_{2}=p_{1}^{2}+p_{2}^{2}+\ldots +p_{N}^{2}\\ \vdots \\ s_{N}=p_{1}{}^{N}+p_{2}^{N}+\ldots +p_{N}^{N} \end{array}\right..$$

In a bid to mitigate the potential overflow and rounding error in computation of Bessel functions, Equation (19) is further modified and formulated in an exponentially scaled form as:

(23) $$\left\{\begin{array}{l} f(x)=e^{\left|ℑ\left(xc\right)\right|+\left|ℑ\left(qh\right)\right|+\left|ℑ\left(qc\right)\right|}\hat{f}\left(x\right)\\ \hat{f}\left(x\right)=x{\hat{J}}_{0}\left(xc\right){\hat{R}}_{1}\left(qc\right)-q{\hat{J}}_{1}\left(xc\right){\hat{R}}_{0}\left(qc\right) \end{array}\right.,$$

where ℑ denotes the imaginary part of a complex value. This scaling method is implemented based on the embedded functions “*besselj* (*n*, *x*, 1)” and “*bessely* (*n*, *x*, 1)” in MATLAB, and gives solutions to *e*^−|^*^ℑ^*^(*x*)|^*J_n_*(*x*) and *e*^−|^*^ℑ^*^(*x*)|^*Y_n_*(*x*) which are efficient in mitigation regarding overflow and reduction in computational accuracy of Bessel functions. The scaled forms of *J_n_*(*x*) and *Y_n_*(*x*) are ${\hat{J}}_{n}\left(x\right)$ and ${\hat{Y}}_{n}\left(x\right)$ whilst ${\hat{R}}_{n}\left(qc\right)$ is the scaled form of *R_n_*(*qc*). ${\hat{R}}_{n}(qc)$ can thus be expressed as:

(24) $${\hat{R}}_{n}\left(qc\right)=\left[{\hat{Y}}_{1}\left(qh\right){\hat{J}}_{n}\left(qc\right)-{\hat{J}}_{1}\left(qh\right){\hat{Y}}_{n}\left(qc\right)\right].$$

In addition, ${\hat{f}}^{\prime }\left(x\right)$, which is the scaled form of *f’*(*x*) can also be derived:

(25) $$f^{\prime }\left(x\right)=\;e^{\left|ℑ\left(xc\right)\right|+\left|ℑ\left(qh\right)\right|+\left|ℑ\left(qc\right)\right|}\hat{f^{\prime }}\left(x\right).$$

In the virtue of the uniform coefficient in Equations (23) and (25), the ratio term, i.e., *f’*(*x*)/*f*(*x*) in Equations (20) and (21) can be replaced by ${\hat{f}}^{\prime }(x)/\hat{f}(x)$. It is noteworthy that: (1) the function ratio in Equations (20) and (21) is essentially the integrand of the loop-integration for finding the eigenvalue in each discretized subarea when applying the Delves and Lyness algorithm in Reference [27]; and (2) by substituting ${\hat{f}}^{\prime }(x)/\hat{f}(x)$ for *f’*(*x*)/*f*(*x*) and taking it as the integrand, the overflow and rounding error in computation can be suppressed. In a bid to predict PMEC responses to the ILC, the efficient computation of the complex roots of *f*(*x*) is realized via the modified Delves and Lyness formula which is written in the scaled form as:

(26) $$s_{n}=\frac{1}{2\pi j}\oint_{C}x^{n}\frac{\hat{f^{\prime }}\left(x\right)}{\hat{f}\left(x\right)}dx=\overset{N}{\underset{i=1}{\sum }}p_{i}^{n}\;\;\;\;n=1,2,\ldots N,$$

(27) $$s_{0}=\frac{1}{2\pi j}\oint_{C}\frac{\hat{f^{\prime }}\left(x\right)}{\hat{f}\left(x\right)}dx=\overset{N}{\underset{i=1}{\sum }}p_{i}^{0}=N\;.$$

The loop-integration can readily be computed with the MALTAB routine “*integral*”. It is further revealed that in Newton–Raphson algorithm for improving the computation accuracy regarding the complex eigenvalues, the ratio term, i.e., *f*(*x*)/*f’*(*x*) can also be replaced with the exponentially scaled form to avoid computational overflow or rounding errors.

It is noted that the accuracy regarding the computation of complex eigenvalues depends also on the discretized subareas in the complex plane. In the event that the number of eigenvalues in each subarea is increased, it becomes more difficult to solve the system of equations shown in Equation (22). Previous investigation has revealed that the computational accuracy for *p_i_* is undermined when the discretized subarea in the complex plane encloses too many eigenvalues [25]. The relatively large number of *N* also brings about the tedious computation of *s_n_* with *n* varying from 1 to *N*. Therefore, it is vital to properly divide the complex plane into the discretized subareas and limit the maximum number of eigenvalues in each subarea. Different from the proposed iteration algorithms to make the subareas denser in the complex plane to reach the limited number of eigenvalues in Reference [28], in this paper characteristics of eigenvalues’ distribution are firstly investigated and utilized to optimize the shape and size of the subarea in the complex plane.

**Figure 2:**
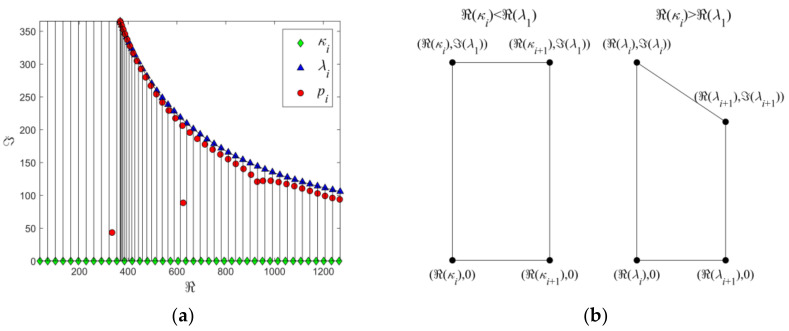
Characteristics regarding the distribution of eigenvalues in the complex plane and defined subareas: (**a**) distribution of eigenvalues *p_i_*, *κ_i_* and *λ_i_* along with the discretized subareas; (**b**) coordinates of the subareas.

When the ILC radius varies from 0 (the defect-free case) to *h* (the wall-thinning case where the ILC radius is considerably larger than the probe size), in the complex plane, the corresponding eigenvalues change from *λ_i_* to *κ_i_*. Due to the continuity in numerical values, the distribution of *p_i_* is thus bounded by *λ_i_* and *κ_i_*. An example regarding the distribution characteristics of eigenvalues *p_i_* in the complex plane for the harmonic case of *ω* = 2000*π* rad/s and the subareas are portrayed in Figure 2. It can be observed from Figure 2a that *p_i_* (red dots) sought in the plane is essentially enclosed by a region with its lower and upper boundaries defined by *κ_i_* and *λ_i_*, respectively. Interestingly, after discretizing the region into a train of rectangular subareas (when *κ_i_* < ℜ(*λ*_1_), where ℜ denotes the real part of a complex value) and trapezoidal subareas (when *κ_i_* > ℜ(*λ*_1_)) with their vertices set as *κ_i_* and *λ_i_*, it can be found that within each resultant subarea the total number of *p_i_* is up to 2. Further investigation indicates that such a finding still holds for the other harmonic cases. The distribution characteristics of eigenvalues provide an advantageous reference to the division of discretized subareas. In light of this, a new approach for discretizing the complex plane is thus proposed. Prior to the computation of the loop integrals in Equations (26) and (27), the complex plane is discretized into a train of subareas in the shapes of rectangles and trapezoids. The coordinates of the vertices defining the contour of each subarea are set as *κ_i_* and *λ_i_*. For the rectangular-shaped and trapezoidal-shaped subareas, the coordinates regarding the vertices of the subarea contour are exhibited in Figure 2b.

It should be pointed out that the proposed approach ensures that there are up to 2 eigenvalues of *p_i_* in each subarea whilst the number of discretized subareas is highly relied on the number of *κ_i_* and *λ_i_*. This makes the subarea discretization free of multiple iteration to obtain denser subareas in the localized complex region where eigenvalues are concentrated and are beneficial to the efficient seeking of all *p_i_* with high computation speed and accuracy. Further investigation also reveals that by taking the *p_i_* sought with the proposed approach as the initial estimates, the number of iterations for refinement of eigenvalues via the Newton–Raphson algorithm for further precision improvement is significantly decreased, which benefits the fast solution to predicted PMEC responses to ILC.

### 2.3. Verification with the Finite Element Modeling

In conjunction with the proposed efficient algorithm for computation of eigenvalues, the established semi-analytical model and formulated closed-form expression of the PMEC signal could be adopted for efficient prediction of testing signals. Prior to theoretical simulations, the semi-analytical model is corroborated by the finite element modeling (FEM). The specimen under PMEC inspection is chosen as a double-layered conductor with cylindrical ILC. The material of each conductor layer is the aluminum alloy. The parameters of the PMEC probe and specimen are listed in Table 1 and Table 2, respectively.

**Figure 3:**
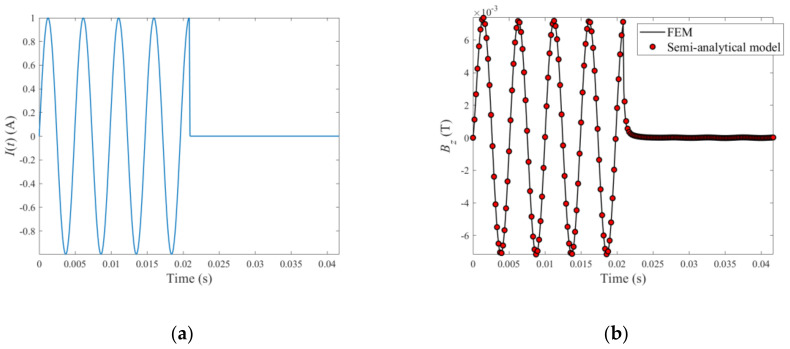
Excitation current and PMEC signals: (**a**) excitation current driving the probe; (**b**) signals predicted by the semi-analytical model and FEM.

The excitation current driving the funnel-shaped PMEC probe and testing signals are exhibited in Figure 3. The maximum amplitude of the excitation current is 1A. It is noted that regarding the excitation-current signal in the pulse-modulation waveform, as per the frequency-selection strategy in Reference [15] the frequencies of modulation wave and carrier wave are set at 24 Hz and 204 Hz, respectively. Different from the excitation signal presented in Reference [15], the carrier wave is truncated by the modulation wave when its amplitude reaches maximum. The comparison of the PMEC signals predicted by the semi-analytical model and FEM is presented in Figure 3b.

It can be observed from Figure 3b that the predicted PMEC signal from the semi-analytical model agrees well with that from FEM. Further analysis implies that compared with the FEM results, the relative error of the computed signal from the semi-analytical model is less than 1%, whilst the simulation time of the semi-analytical model is 10~15 times faster than FEM, indicating the superiority in terms of efficiency regarding the established semi-analytical model to FEM. 

## 3. Theoretical Simulations and Discussion

**Figure 4:**
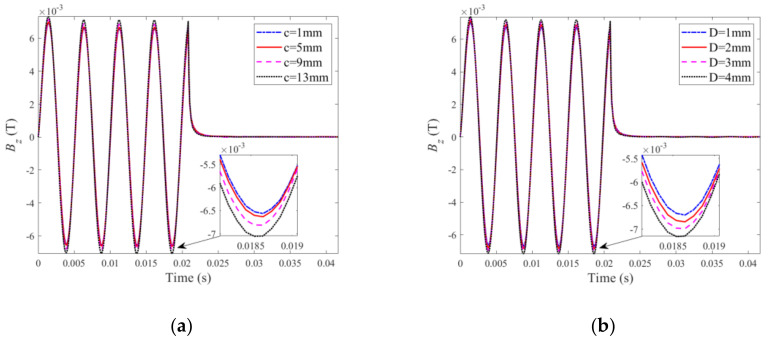
Testing signals predicted by the semi-analytical model: (**a**) ILCs with various radii; (**b**) ILCs with various depths.

Following verification, the PMEC responses to ILCs with different radii and depths are scrutinized through theoretical simulations based on the established semi-analytical model. For the ILC scenarios of various radii (*c* = 1~15 mm with *D* fixed at 4 mm) and depths (*D* = 1~5 mm, while *c* = 15 mm), the predicted PMEC signals are exhibited in Figure 4.

**Figure 5:**
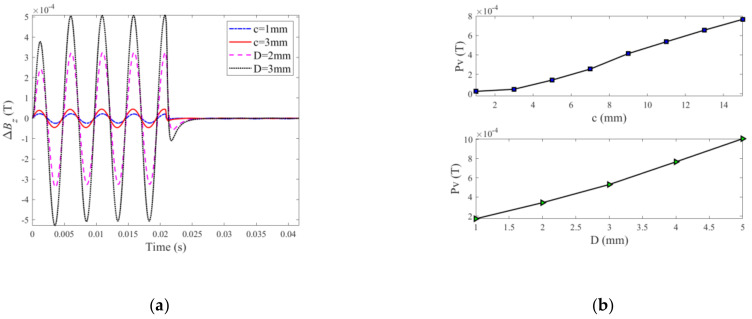
Difference signal and correlations of Pv: (**a**) difference signals with various radii and depths of ILC; (**b**) correlations of Pv with the radius and depth of ILC.

It can be seen from Figure 4 that the signal amplitude increases as the ILC size in terms of the radius (shown in Figure 4a) and depth (shown in Figure 4b) rises. The reasoning lies in the fact that the increase in the ILC volume enhances the perturbation of eddy currents induced within the specimen, causing the drop in the eddy-current-induced field and thus the increase in the net magnetic field. Further signal processing is conducted by subtracting the reference signal for the defect-free case from the defect signals for different ILC scenarios, giving the so-called difference signals. The peak value (Pv) is subsequently extracted from the difference signal. The correlations of Pv with the radius and depth of ILC are presented in Figure 5.

As can be observed from Figure 5b, amplitude of difference signals will rise with the increase of radius and depth of ILC. In Figure 5b, Pv which is the extreme value extracted from the PMEC difference signal is closely associated with the sizing parameters of the ILC with a positive correlation. It is directly proportional to either radius or depth of the ILC, indicating that more eddy currents are perturbed in the presence of the ILC with the increasing size. Therefore, Pv is utilized as the signal feature for detection, imaging and evaluation of ILC in layered conductors.

## 4. Experiments

**Figure 6:**
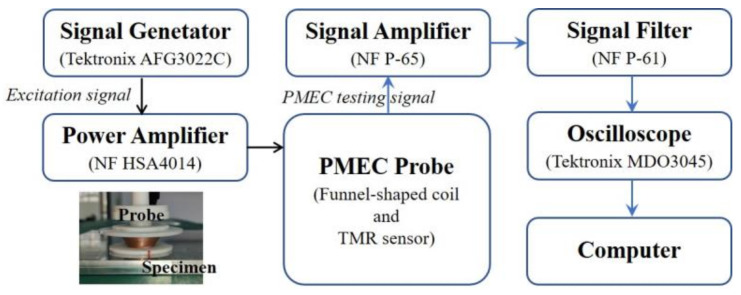
Schematic illustration of the PMEC inspection system (inset: the funnel-shaped probe and testing specimen).

In parallel to the theoretical investigation, a PMEC inspection system has been built up for detection, imaging and assessment of ILC in stratified conductors. The schematic illustration of the PMEC system along with the picture of the fabricated funnel-shaped probe and testing specimen is portrayed in Figure 6.

The parameters of the probe and specimen are same as those tabulated in Table 1 and Table 2. The in-house PMEC probe generates the incident field with the funnel-shaped coil whilst a Tunnel Magneto-Resistance (TMR) sensor (MultiDimension TMR 2505) deployed at the bottom center of the coil is used to pick up the *z*-component of the net magnetic field. The excitation signal driving the probe is in pulse-modulation waveform with the frequencies of modulation wave and carrier wave set at 24 Hz and 204 Hz, respectively, whilst its maximum amplitude is 0.3 A.

**Figure 7:**
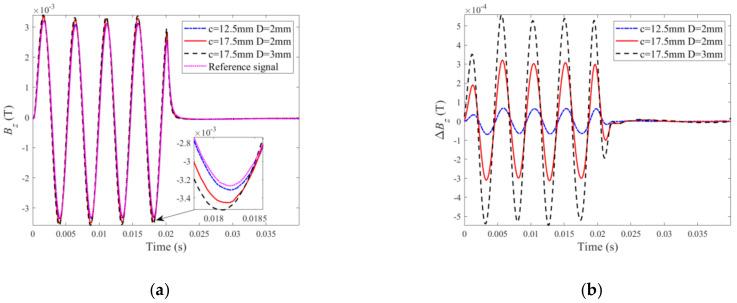
Experimental signals for different ILC parameters: (**a**) testing signals; (**b**) difference signals.

The layered specimens adopted in experiments consist of two aluminum-alloy layers whose conductivity and relative permeability are 34 MS/m and 1, respectively. Flat bottom holes imitating ILCs with different radii (from 12.5 mm to 17.5 mm) and depths (from 1 mm to 4 mm) are fabricated at the interface of the layers and reside at: (1) the back surface of the upper layer for ILC Scenario #1; and (2) the surface of the bottom layer for ILC Scenario #2. During experiments, the probe is scanned over the specimen by a scanning table with the spatial resolution of 1 mm whilst the lift-off of the probe is fixed at 2 mm. The reference signal is obtained when the probe is right over the defect-free area of the specimen. Difference signals are acquired by subtracting the testing signal at each scanning position from the reference signal. The experimental signals and derived difference signals when the PMEC probe is placed right over the center of each ILC are portrayed in Figure 7.

It can be observed from Figure 7a that the amplitude of the testing signal increases as the size of ILC is increased due to more perturbation of eddy currents by ILC whilst the magnitude of the difference signal presented in Figure 7b rises when the radius and depth of ILC are increased. This is consistent with findings from the theoretical investigation. It is also noticeable that the amplitudes of the testing signals are lower than those for the back-surface corrosion in a conductive plate (analogous to a single-layer conductor). This is due to the fact that more eddy currents are induced within the multilayered conductor, and thus the eddy-current-induced field opposing the incident field becomes stronger, resulting in the decrease in strength of the net magnetic field.

**Figure 8:**
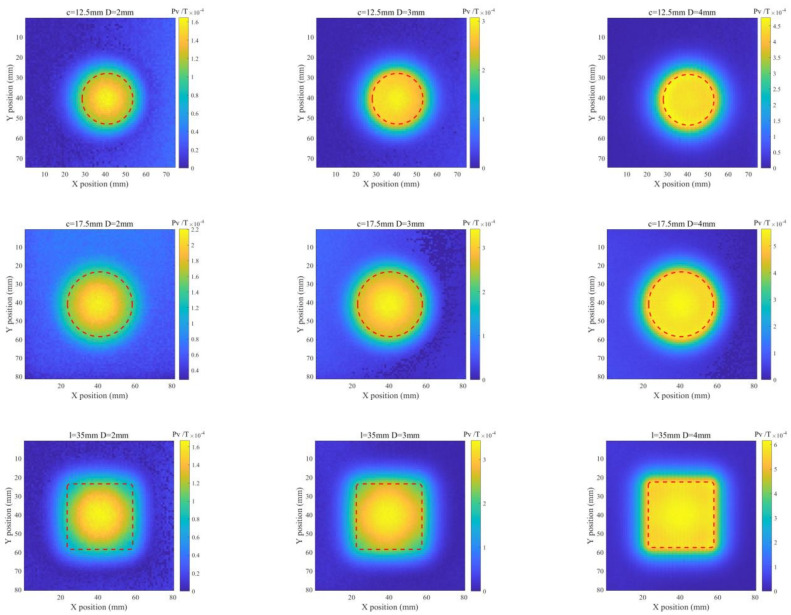
Imaging results for ILC Scenario #1 (where *l* denotes the length of the square-shaped ILC).

**Figure 9:**
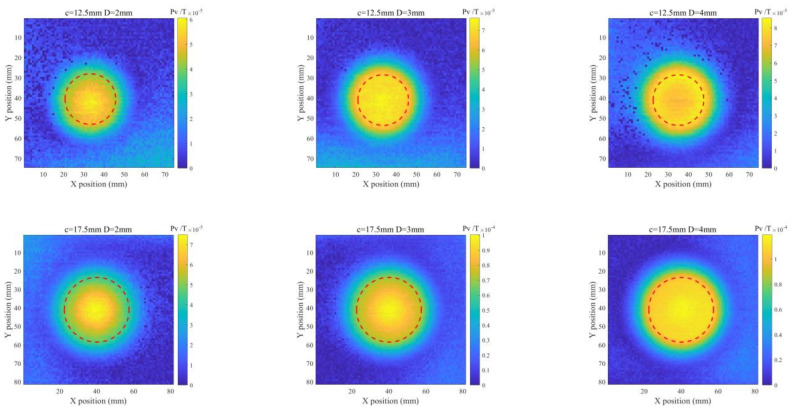

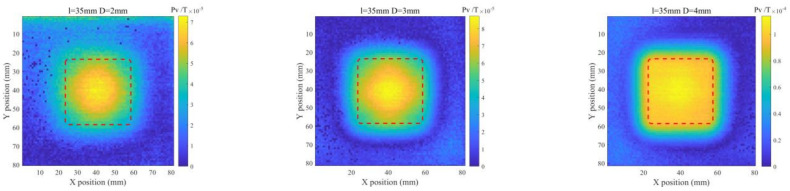
Imaging results for ILC Scenario #2.

It is also noticed from Figure 7b that Pv of the difference signal is directly proportional to the ILC size. Therefore, Pv extracted from the difference signal is subsequently used for ILC imaging. With all Pvs obtained at scanning positions, the Pv-based images of ILCs with different radii and depths are produced. The imaging results for various ILC scenarios are exhibited in Figure 8 and Figure 9 against different sizes of ILCs in either circle or square shape for ILC Scenarios #1 and ILC Scenarios #2. It is observed from Figure 8 and Figure 9 that the position of each ILC can be readily localized by using the imaging results along with the indication regarding the ILC size.

**Figure 10:**
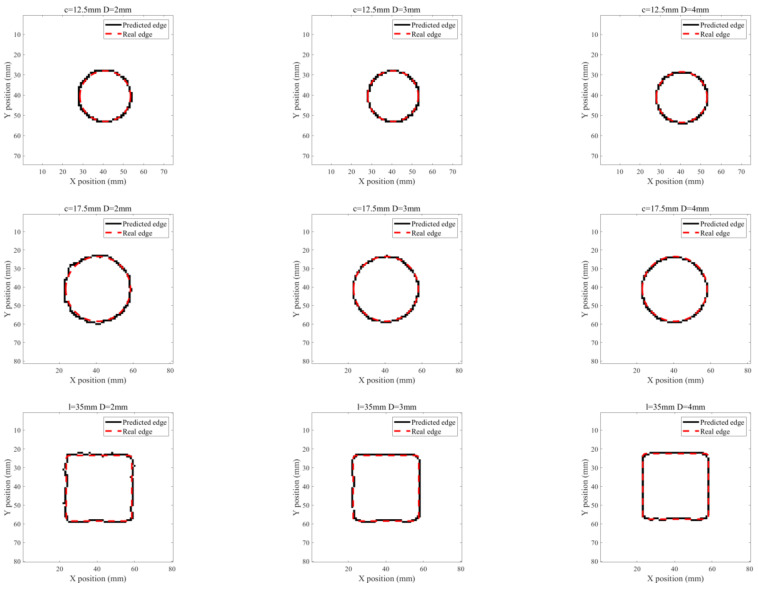
Edge-identification results for ILC Scenario #1.

**Figure 11:**
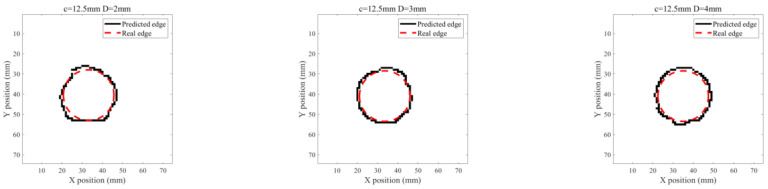

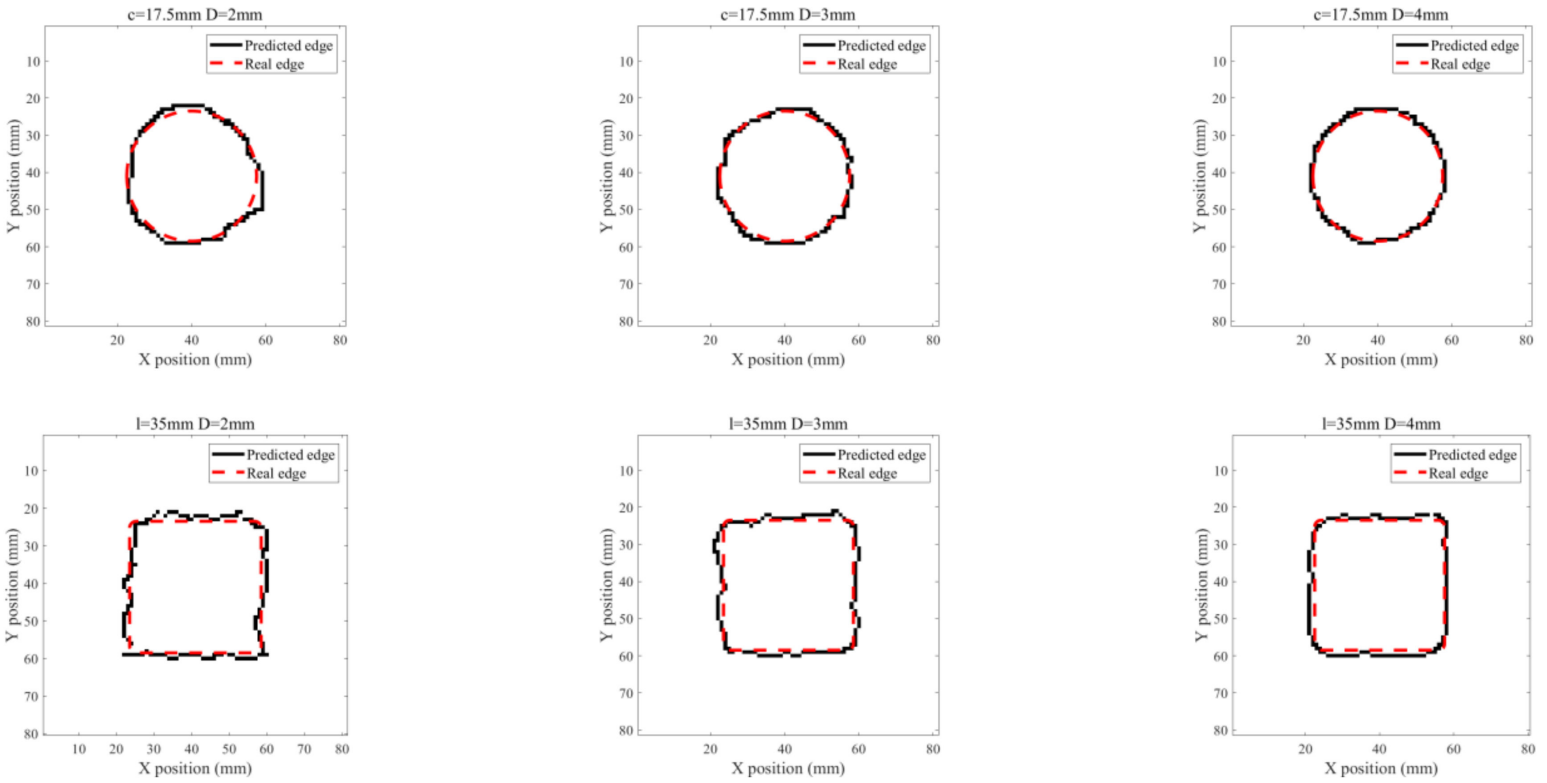
Edge-identification results for ILC Scenario #2.

Based on the acquired ILC images, further investigation is carried out to analyze the feasibility of PMEC for evaluation of ILCs in terms of the ILC opening size and depth. A Canny-algorithm-based edge recognition method [16] is applied for identification of the ILC edge with Pv-based images. The processed image results are shown in Figure 10 and Figure 11. It can be seen from Figure 10 and Figure 11 that the identified ILC edge agrees well with the real ILC opening profile. Following this, the ILC opening size is estimated based on the identification results. The estimation results of the ILC opening size for ILC Scenarios #1 and ILC Scenarios #2 are listed in Table 3 and Table 4. It is noticeable from Table 3 and Table 4 that the approximated area of ILC opening has good agreement with the actual size. The maximum of relative errors is less than 10%.

**Figure 12:**
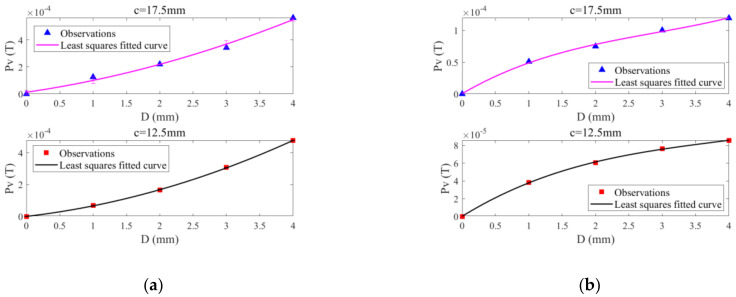
Pv against the ILC depth for: (**a**) ILC Scenario #1; (**b**) ILC Scenario #2.

Based on the identification results regarding the opening of each ILC, the center of every ILC is determined, and the Pv corresponding to the ILC center is extracted from the Pv-based image in order to establish the correlation between the Pv and ILC depth. The obtained correlation curves are shown in Figure 12. As can be observed from Figure 12 that for every ILC scenario, the Pv has a monotonic correlation with the ILC depth whilst Pv rises with the ILC depth increased. This agrees with the finding from the simulations. Both theoretical simulation and experiment indicate that the ILC depth can readily be evaluated by using the correlation curve. The ILC radius can be assessed by using the processed ILC image with the Canny-algorithm-based edge recognition method. The feasibility of PMEC for the detection, imaging and evaluation of ILC in stratified conductors is confirmed.

## 5. Conclusions

In an effort to intensively investigate the feasibility of PMEC for the detection, imaging and evaluation of ILCs in layered conductors, in this paper a semi-analytical model for the efficient prediction of PMEC responses to ILC has been established along with the resulting formulation of the closed-form expression of the PMEC testing signal. More efforts have been given to the reliable computation of eigenvalues for the ILC region. The exponentially scaled form of the integrand in the loop integral is deduced in a bid to mitigate the overflow and rounding errors in computation. An approach for discretizing the complex plane for an efficient solution to eigenvalues has been proposed. Following the establishment of the semi-analytical model, the characteristics of PMEC responses to ILC and correlation of the PMEC signal feature with the sizing parameter are investigated through theoretical simulations. It has been found that the Pv extracted from the difference signal has a monotonic relation with the ILC size. Therefore, it is used for ILC imaging.

In conjunction with the finding from simulations, the feasibility and applicability of PMEC for imaging and evaluation of ILC in a stratified conductor are further investigated via experiments. It can be noticed that the Pv-based ILC image implies the position and size of the ILC. The processed image with the Canny-algorithm-based edge recognition method can be adopted for approximation regarding the opening area of the detected ILC. Complying with the finding from simulations, the correlation of Pv with the ILC depth is monotonic, and the resultant correlation curve is applicable for the estimation of ILC depths.

Following the affirmation regarding the feasibility of PMEC for the imaging and evaluation of ILCs, further work involves: (1) investigation regarding the advantages of PMEC over other NDE methods such as PEC in particular; (2) imaging of ILCs in irregular shapes/profiles; and (3) 3D reconstruction of ILCs in layered conductors.

## Figures and Tables

**Table 1:** Parameters of the PMEC probe.

Symbol	Quantity	Value
*r*_1_	Inner radius of the coil bottom	8.0 mm
*r*_2_	Inner radius of the coil top	14.8 mm
*δ*	Coil radial thickness	0.8 mm
*z*_1_	Lift off	2.0 mm
*z*_2_	Position of the coil top	12.1 mm
*N_coil_*	The number of coil turns	205
(*r*, *z*)	Sensor location	(0, 1) mm

**Table 2:** Parameters of the specimen.

Symbol	Quantity	Value
*h*	Specimen length	100 mm
*d*_3_ = 2*d_Layer_*	Specimen thickness	8.0 mm
*σ*	Specimen conductivity	34.0 MS/m
*μ_r_*	Specimen relative permeability	1
*d*_1_	Upper boundary of ILC	2.0 mm
*D*	ILC thickness	4.0 mm
*c*	ILC radius	15.0 mm

**Table 3:** Assessment results regarding ILC opening areas for ILC Scenario #1.

ILC Radius/Length	ILC Depth	Estimated Area	Actual Area	Relative Error
*c* = 12.5 mm	2 mm	471 mm^2^	490.87 mm^2^	4.05%
	3 mm	449 mm^2^	490.87 mm^2^	8.53%
	4 mm	456 mm^2^	490.87 mm^2^	7.10%
*c =* 17.5 mm	2 mm	957 mm^2^	962.11 mm^2^	0.53%
	3 mm	910 mm^2^	962.11 mm^2^	5.42%
	4 mm	903 mm^2^	962.11 mm^2^	6.14%
*l* = 35 mm	2 mm	1196 mm^2^	1225 mm^2^	2.37%
	3 mm	1181 mm^2^	1225 mm^2^	3.59%
	4 mm	1157 mm^2^	1225 mm^2^	5.55%

**Table 4:** Assessment results regarding ILC opening areas for ILC Scenario #2.

ILC Radius/Length	ILC Depth	Estimated Area	Actual Area	Relative Error
*c* = 12.5 mm	2 mm	526 mm^2^	490.87 mm^2^	7.16%
	3 mm	519 mm^2^	490.87 mm^2^	5.73%
	4 mm	539 mm^2^	490.87 mm^2^	9.81%
*c =* 17.5 mm	2 mm	964 mm^2^	962.11 mm^2^	0.20%
	3 mm	954 mm^2^	962.11 mm^2^	0.84%
	4 mm	950 mm^2^	962.11 mm^2^	1.26%
*l* = 35 mm	2 mm	1224 mm^2^	1225 mm^2^	0.08%
	3 mm	1244 mm^2^	1225 mm^2^	1.55%
	4 mm	1260 mm^2^	1225 mm^2^	2.86%

## Data Availability

Not applicable.
